# Policies and programs for road safety in developing India

**DOI:** 10.4103/0974-2700.41790

**Published:** 2008

**Authors:** Nishi Mittal

**Affiliations:** Division of Environment and Road Traffic Safety, Central Road Research Institute, New Delhi, India

Globalization has brought India into the forefront of progress. This development has provided a substantial economic stimulus to enhance progress across India which includes enabling people to afford cars and increase their movement on the roads. Various multinational companies including motor vehicle manufacturers have created establishments in India. The increased usage of the cars has enhanced the need for developing the infrastructure where these motor vehicles can move safely. Developing safe roads which connect destinations and cities is a key foundation to infrastructural development in a country where the population is more than 1 billion. This commentary reviews various aspects of road safety in heavily populated and developing India where motor vehicle trauma is a modern epidemic.

## GLOBAL ROAD SAFETY SITUATION

Road deaths and injuries are a global problem of massive proportions. Of all the systems that people have to deal with on a daily basis, road transport is the most complex and the most dangerous. In recent years, some important and major studies on the subject of road accidents and fatalities carried out by World Bank, World Health Organization (WHO), Transport Research Laboratory (TRL), and others have highlighted the growing significance of road crashes as a cause of death particularly in developing and transitional countries.

In the year 1999, between 750,000 and 880,000 people died due to road traffic crashes,[1] while the World Bank in its study in 1998[2] estimated a total of 1 million deaths due to road traffic crashes. This itself demonstrates the severity of the problem worldwide.

According to WHO,[3] every day around the world, almost 16,000 people die from all types of injuries. Injuries represent 12% of global burden of disease, the third most important cause of overall mortality, and main cause of overall mortality among 1 to 40 years old. According to WHO data, deaths from road traffic injuries account for around 25% of all deaths from injury.

WHO and World Bank in its report[3] released on World Heath Day, 2004 estimated the number of people killed in road traffic crashes at almost 1.2 million, while the number of injured as high as 50 million (WHO, 2004). According to WHO, road traffic injuries are the leading cause of death by injury worldwide (20.3% of all deaths from injury). Road traffic injuries rank second to HIV/AIDS as the leading cause of ill-health and premature death for adult men aged 15-44 years.

The study by WHO in 1996, ‘Global Burden of Disease’ showed that in 1990, out of 10 causes of deaths and injuries, road crashes were ninth on the list. However, forecast for 2020[4] shows that the road crashes are expected to move up to third place in terms of disability adjusted life years' (DALYs). The DALY is an indicator of the time lost by an individual in living with a disability and the time lost due to premature death. Jacob and Aeron-Thomas[1] suggested that for 2010 the likely range of global road deaths will be between 900,000 and 1.1 million and between 1 million and 1.3 million in 2020.

It is estimated that without additional efforts and new initiatives, the total number of road traffic deaths worldwide and injuries is forecast to rise by some 65% between 2000 and 2020 and in low-income and middle-income countries the deaths are expected to increase by as much as 80%.

The majority of road crash deaths, about 70%, occur in developing countries. Sixty-five percent of deaths involve pedestrians and 35% of pedestrian deaths are children. Over 10 million are crippled or injured each year. It has been estimated that at least 6 million more will die and 60 million will be injured during the next 10 years in developing countries unless urgent action is taken.

The majority of road crash victims (injuries and fatalities) in developing countries are not the motorized vehicle occupants, but pedestrians, motorcyclists, bicyclists, and nonmotorized vehicles (NMV) occupants. In high-income countries, deaths among car occupants continue to be predominant, but the risks per capita that vulnerable road users (VRUs) face are high.

## ROAD SAFETY SCENARIO IN DIFFERENT REGIONS OF THE WORLD AND INDIA

The study sponsored by Global road safety partnership (GSRP) provides a detailed summary of the road safety situation in the individual regions of the world.[1] The differences within the regions however, are often as wide as those between them.

From Table 1, it can be found that Asia/Pacific region accounts for a total of 44% of the road fatalities although it has only 16% of the total vehicular ownership of the world and 54% of the world's total population. India has just 1% of the world's vehicle population, but accounts for nearly 10% of the road traffic fatalities, while highly motorized countries (HMC) having a total of 60% of the world's motor vehicles account for only 14% of the total road fatalities. As may be seen from Table 2, South-East Asia Region had the maximum share of road safety problems in the world.[5]

**Table 1:** Distribution of global deaths and licensed vehicles

Region	Global percentage of		
	
	Road fatalities	Vehicles	Population
Highly motorized countries	14	60	15
Asia/Pacific	44	16	54
Central/Eastern Europe	12	6	7
Latin America/Caribbean	13	14	8
Africa	11	4	11
Middle East/North Africa	6	2	4
Total	100	100	100

**Table 2:** Distribution of road traffic deaths and mortality rates, by WHO region* and income group* (high and low/middle), 1998

Country:	AFR	AMR		EMR	EUR		SEAR	WPR		World
							
Income Group		HICs	LMCs		HICs	LMCs		HICs	LMCs	
Total RT Deaths (000)	170	49	126	72	66	107	336	25	220	1171
% of global RT deaths	14.5	4.2	10.8	6.1	5.6	9.1	28.6	2.1	18.8	100
RT deaths per 100,000	28.2	16.1	25.3	15.2	16.8	22.4	22.6	12.6	15.5	19.9
% of all deaths due to RTI	1.8	1.9	4	1.9	1.7	2	2.5	1.7	2.1	2.2

AFR = AFRICAN REGION AMR = AMERICAN REGION, HIC = HIGH INCOME COUNTRIES LMC = LOW AND MIDDLE COUNTRIES EMR = EASTERN MEDITERRANEAN REGION, EUR = EUROPEAN REGION. SEAR = SOUTH-EAST ASIAN REGION, WPR = WESTERN PACIFIC REGION

**Figure 1:**
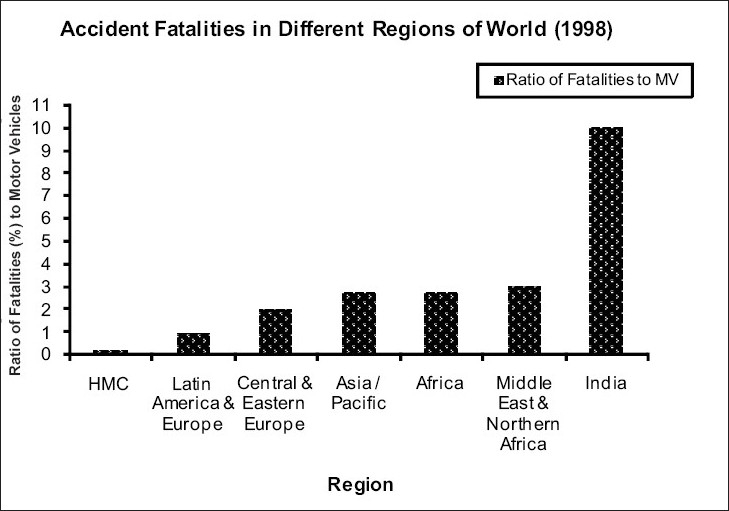
Accident fatalities in different regions of the world (1998)

**Figure 2:**
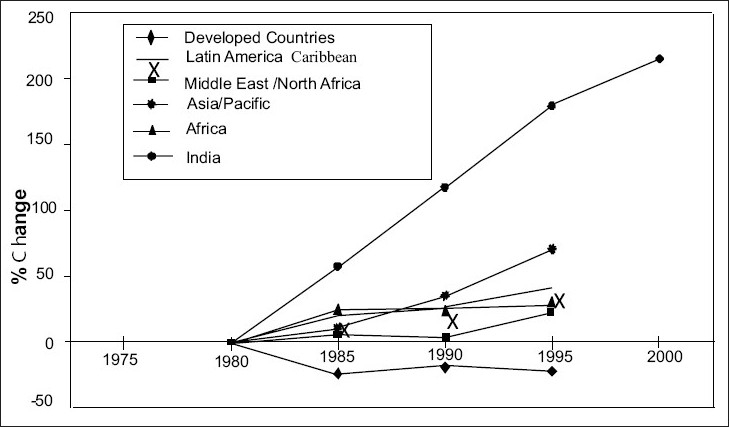
Growth of road fatalities in different regions

The basic reason for comparatively much better situation of HMC can be attributed to policies and programs adopted and carried out there to improve road safety. Figure 1 shows ratio of fatalities to motor vehicles and it is obvious as to how badly India stands vis-à-vis to all regions of the world. Figure 2 further reinforces the same fact. It shows that road safety situation in India is going from bad to worse every year.

One of the most important differences between developed and developing regions is tht over the last 10 years or so the number of deaths taking place actually fell by about 10% in Western Europe and North America, while in the Asia/Pacific and Latin America regions road deaths continued to rise. Results also show that the highest fatality rates (deaths per 10,000 motor vehicles) worldwide occur in African countries, particularly Ethiopia, Uganda, and Malawi while fatality risk (deaths per 100,000 population) is highest in a disparate group of countries including Thailand, Malaysia, South Africa, and Saudi Arabia.

It should be emphasized that pedestrians are a particularly high-risk group throughout Africa and Asia as well as the Middle East. Car occupant casualties dominate in developed countries and are much more common in the Latin America/Caribbean region. Road traffic injuries involve issues of social equity, having a disproportionate impact on the poor in developing countries where most victims are VRUs such as pedestrians, children, cyclists, and passengers. As poorer members of society have less access to medical services, their chances of recovery after crashes are relatively lower.

There are many reasons for the differences between countries and certainly between developed and developing countries. These might include differences in culture, education, socioeconomic characteristics, education, road user behavior, driver training, vehicle mode, and use (e.g., trucks used for transportation of people and general overloading of public transport vehicles), vehicle and road condition, vehicle mix and composition, and a higher use of rural and inter-city roads by pedestrians and slow-moving vehicles.

Road traffic injuries also have disproportionate effect on young people. These accidental deaths come at any age without warning. Over 50% of deaths worldwide occur among young adults aged between 15 and 44. Males are almost three times more vulnerable than females: in 2002, the rates were 27.6 per 100,000 males and 10.4 per 100,000 females. As people in the age groups that are most economically active are most affected by road crashes, there is an increased burden on poorer countries attempting to tackle poverty and raise levels of economic growth. As per the Working Group on Road Accident and Injury Prevention and Control of Planning Commission, India[6] the total cost of road accidents amounted to over 2% of gross domestic product.

Risk on the roads is at least five times the average risk elsewhere in everyday life. The impact in terms of emotional and financial stress is enormous. Poverty, depression, physical illness, and suicide are common consequences. The happening of road crashes also leaves substantial impact on families and friends, and community in general. Reduced social interaction and cohesion is the result forcing people to remain indoors. In many countries, it has resulted in forcing people to remain indoors. It has also resulted in more sedentary life styles, with consequent health effects such as obesity and cardiovascular diseases.

## COMPARATIVE EFFORTS AND SUCCESSES IN IMPROVING ROAD SAFETY IN INDIA AND ELSEWHERE

When accidents became increasingly common throughout the physical space of road networks, industrialized countries began to realize they were facing a new phenomenon. The first major change was seeing the accident as a man-made problem. Road traffic crashes were no longer viewed with a “fatalistic” attitude, as a question of fate, but as a consequence of human actions. Therefore, they could be prevented. The second major change that followed was seeing the accident as a public health problem, therefore deserving special attention from the state. This status was reinforced by two combined features: traffic fatalities increased to high levels and became as damaging as contemporary illnesses such as cardiovascular disease or cancer, causing large social and economic losses to society. The mobilization of society and the state around this issue led to new combined efforts in dealing with accidents. Major comprehensive, interdisciplinary programs were implemented in several developed countries from the 1950s to the 1970s, with a remarkable effect in reverting the upward tendency in the number and severity of traffic accidents.

Unlike developed countries, the accident problem in developing countries has not yet assumed the status of a social issue. Public acknowledgement of the problem is still divided into conflicting views, ranging from the “fatalistic” to the “unavoidable-cost-of-development” approaches. Therefore, policy decisions have entailed different and sometimes conflicting actions, pursued independently by various public agencies, with poor outcomes.[7]

**Figure 3:**
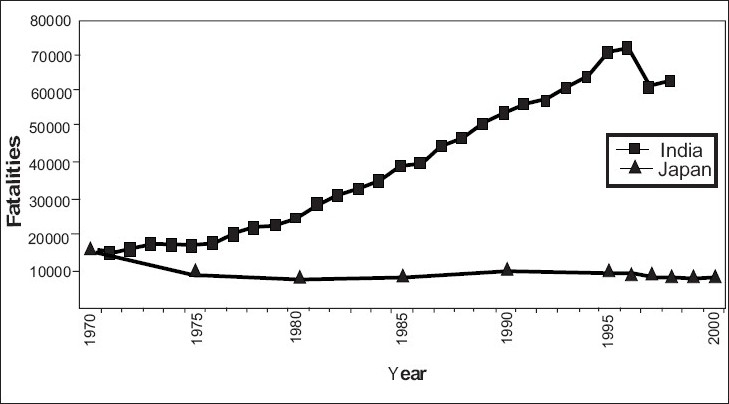
Comparative fatality trends: India and Japan

The year of 1970 was seen as one of the worst years for Japan in terms of road traffic fatalities, the fatalities recorded in India during the same year were almost the same as that of Japan. India recorded about 15,000 fatalities in the year 1971 while Japan had around 17,000 fatalities in 1970. Japan analyzed the severity of the problem and took up various road safety initiatives including its first road safety plan and hence was not only able to restrict the fatalities but also reduce them [Figure 3]. On the other hand, fatalities in India have grown in an alarming manner since then due to lack of adequate and planned road safety initiatives.

**Figure 4:**
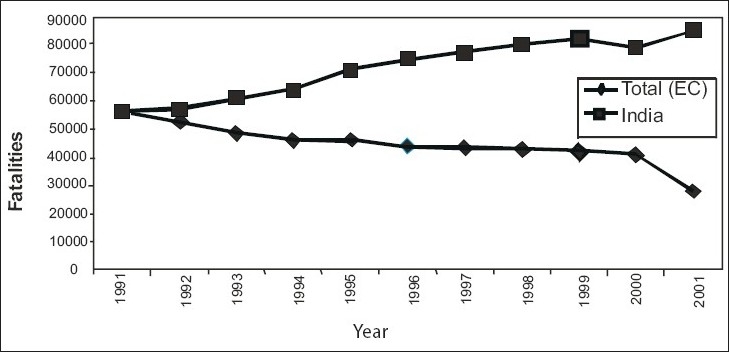
Comparative fatality trends: India and European countries

In the year 1991, the total number of fatalities recorded in India was 56,400 while the European Commission (combined) recorded 56,027 fatalities during the same year [Figure 4]. Therefore, it can be seen that there was hardly any difference in the total number of fatalities between India and European Commission (EC) in the year 1991. European countries, understanding the severity of the problem continued with the road safety policies they had implemented earlier and introduced some new policies. The result was emphatic as evident from [Figure 4]. On the one hand, where the European countries have been able to reduce the number of fatalities in 10 years by almost 50%, India on the other hand has witnessed increase in the number of fatalities by almost 50% thereby raising serious doubts about safety on roads in India. India's increase is explained by the faster growth in motorized vehicles and population compared to European countries. The graph suggests that India is doing very badly compared to Europe. The noticeable point to be observed from is that each of the 15 countries was able to reduce the number of fatalities during the last decade (1991-2000).

Moreover, the reduction in the number of fatalities was not only in decadal terms, but also consistently on yearly basis for each of the EC countries.

**Figure 5:**
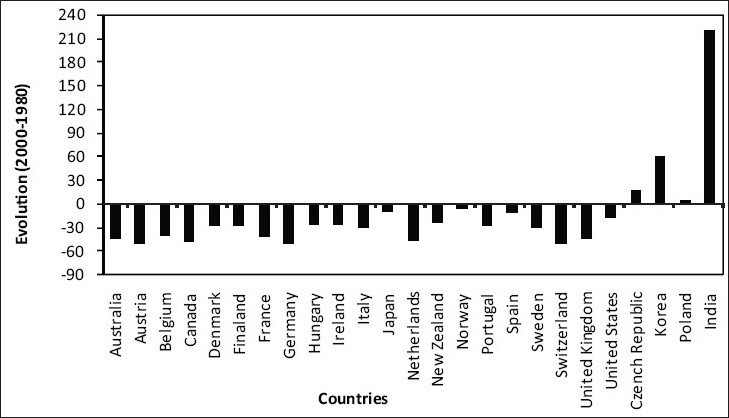
Changes in fatalities in selected countries (2000-1980)

Developed countries such as United Kingdom and United States have taken the issue of road traffic crashes very seriously, initiating certain policies and actions for its improvement. These programs and policies have now stabilized or in fact reduced fatalities in these countries [Figure 5]. Other countries including the Asian, Latin American, African, and the Middle East have not been able to either stabilize or reduce the fatalities. On the other hand, in these countries the fatalities have only increased with time [Figure 2] and the Asian countries have recorded the highest growth rate in fatalities due to road traffic crashes.

As countries develop death rates usually fall especially for diseases that affect the young and result in substantial life-years lost. Deaths due to traffic accidents are a notable exception: the growth in motor vehicles that accompanies economic growth usually brings an increase in road traffic accidents. Table 3 highlights the increasing importance of the problem in several developing countries. For example, between 1975 and 1998, road traffic deaths per capita increased by 44% in Malaysia and by over 200% in Colombia and Botswana.

**Table 3:** Change in traffic fatality rate (deaths/10,000 persons), 1975-1998

Country	% Change (1975-1998)	Country	% Change (1975-1998)
Canada	-63.4	Taiwan	-32.0
Hong Kong	-61.7	United States	-27.2
Finland	-59.8	Japan	-24.5
Austria	-59.1	Malaysia	44.3
Sweden	-58.3	Sri Lanka	84.5
Israel	-49.7	India	184.8
Belgium	-43.8	Lesotho	192.8
France	-42.6	Colombia	237.1
Italy*	-36.7	China	243.0
New Zealand	-33.2	Botswana†	383.8

* % CHANGE (1975-1997),

† % CHANGE (1976-1998)

The situation in high-income countries is quite different. Over the same period, traffic fatalities per person decreased by 60% in Canada and Hong Kong, and by amounts ranging from 25% to 50% in most European countries. This reflects a downward trend in both the fatality rate (deaths/population) and in fatalities per kilometer traveled that began in most organization for economic co-operation and development countries in the early 1970s and has continued to the present.

These patterns are not surprising. The traffic fatality rate (fatalities/population) is the product of vehicles per person (V/P) and fatalities per vehicle (F/V). How rapidly fatality risk grows depends, by definition, on the rate of growth in motorization (V/P) and the rate of change in fatalities per vehicle (F/V). In most developing countries, over the past 25 years, vehicle ownership grew more rapidly than fatalities per vehicle fell. The experience in industrialized countries, however, was the opposite; vehicles per person grew more slowly than fatalities per vehicle fell.

## SAFETY CONSCIOUS PLANNING

Among the countries that have been most successful in reducing the number of crashes, the main factors seem to include:

- A political commitment, across all ministries concerned, to deal with road accidents
- A broad strategy, involving education, engineering and enforcement to deal with accidents
- A clear vision and numerical targets for reductions in the different categories of accidents
- A concrete plan with specific measures for implementation and enforcement
- Institutional co-ordination within and between different levels of government and with private actors
- Careful and critical evaluation of measures and their effectiveness

It is obvious that above-mentioned initiatives have not been taken in India. The culture of road safety develops gradually and there are three distinct stages of such a development as outlined in Table 4.

**Table 4:** Various development stages of road safety

Development stage and level of awareness	Accident data availability	Organizations involved	Government's commitment
*Stage-I*			
Little safety awareness	Data system absent or very primitive Little is known about trends or road users at risk	No one working specifically on road safety matters	General interest by the government is low
*Stage-II*			
Road safety interest beginning to emerge	Accident data are sparse	Occasional road safety pressure groups	Government accords little priority although aware of the problem
	Some university research may be underway	Some interest may be shown by media	Any road safety work is fragmented and un-coordinated
*Stage-III*			
Enlightenment about road safety among various stakeholders	Improved data system gets established	Core of people specialized in safety planning/operations	Government recognizes need for assistance and actions
	Identification of black-spots and road user gropus at risk	Road safety research is being undertaken Media active in pushing action	Improvement of diving test and vehicle examinations
		Road safety Councils at national, regional and local levels	Improvement in institutional arrangements and funding mechanisms

From the study of the table, it can be seen that although India has crossed Stage-II it is only just at the entry point of Stage-III phase. It has yet a long way to go to fulfill the salient characteristics of Stage-III to hope for significant breakthrough in the area of road traffic safety. Many countries have been able to achieve a better safety record in last 30 years or so. The conscious approach adopted by these countries to address the road safety aspects for planning, engineering, etc. has been the main reason for their success. Initially the developed countries tried hard to reduce the number of accidents through the policies and plans, but after summing the situation they realized it was more fruitful to reduce the severity of accidents than decreasing the actual number of accidents. The methods undertaken by these countries include road safety audits, accident black spot reduction, speed regulation, etc. It was also felt that taking account of road safety problems during the planning stage itself reduces the burden of expensive solution later on.

## NEED FOR ROAD SAFETY POLICIES

Road crashes are preventable. Significant number of road deaths and injuries are not a fundamental law of nature or an inevitable result of motorization. Countries that have been successful in improving their road safety like Sweden, New Zealand, Netherlands, Japan, and others have achieved success due to adoption of road safety action plans. Adoption of an appropriate road safety policy was the main driving force for such a major reduction in road fatalities in these countries. It was recognized that an appropriate road safety policy was one of the essential elements of a well-balanced overall transport policy and a public health policy. Some developing countries are also now demonstrating that significant reductions can be achieved via implementation of appropriate policies. For example, in Hungary, a 39% decrease in the number of accidents on roads happened between 1990 in comparison to 1994, largely due to the adoption of road safety policies and programs.

**Figure 6:**
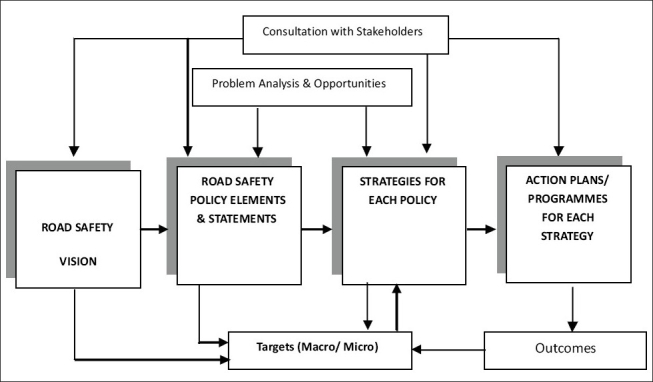
A schemata for developing road safety policy and programming

Governments and road safety organizations act at many levels to diminish the risk of road crashes. This complexity demands the adoption of some method of planning. The advantages of planning such a complex process are quite evident: the goals become apparent to all parties involved; it simulates effective and efficient countermeasures to identified problem areas; it enables all relevant parties to deliver their contributions in a timely and co-operative manner and feedback to the plan allows easy modifications. Such road safety plans necessarily contain a vision statement, policy guidelines, strategies, and specific actions to be undertaken. A schemata for developing road safety policies and programs is shown in Figure 6. It is evident from the figure that vision, policies, strategies, and action plans form the hierarchy of road safety planning, with important inputs in the shape of problem analysis, target setting, and consultations with all important stakeholders at different stages of project formulation for any jurisdiction, e.g. national, state, or local level.

### Defining road safety policies

Road safety policy is nothing but a statement of various steps to be undertaken to achieve the visualized targets, set in this field to tackle identified safety issues. Every country wishes to make its roads as safe as possible, i.e. it visualizes that in future road accidents will be either zero or the minimum possible. However, state vision cannot be accomplished unless followed by suitable timeframe and fund allocation. Further, these stated targets cannot be accomplished unless and until accompanied by action plans suited to the requirements of the country within its political, economic, and social setup. All this requires identification of the problems, determination of appropriate measures and preparation of a well-justified, prioritized, time, and coordinated program of selected activities.

### Vision and philosophy for road safety policies

In recent years, visions have been developed in a number of countries for improving road safety [Table 5]. The purpose of a vision is to ensure that road safety gains a prominent place in transport policy and decision-making processes. A vision can also raise public interest and create support in the population and among public and private organizations for road safety improvement. Politically accepted national long-term visions reflect a societal desire to make a serious commitment to substantially improving road safety. Such visions are practical and easy to use at regional and local levels too.

**Table 5:** Examples of the vision statements of various countries

Country	Vision statements
Australia	Safe road use for the whole community
Austria	To have a road safety level that is comparable to the levels found in the top third countries in the European Union.
Canada	To have the safest roads in the world
Denmark	One accident is too many
Great Britain	Tomorrow's roads: safer for every one
Netherlands	Sustainable road safety
New Zealand	To create a safety culture in New Zealand that delivers land transport safety outcomes that achieve world best practices
Sweden	Vision zero

Though in Sweden, in 1996-1997 around 540 people were killed, it adopted ‘*Vision Zero*’ in 1997 to imply a widespread commitment from the society to give priority to prevent the worst consequences of road accidents. Similarly, Netherlands adopted vision ‘*Sustainable Road Safety*’ in 1991 and it has lead to the creation of new design principles. Regional pilot projects have been launched to implement the new strategies. The projects are supported at the national level.

## TARGET SETTING FOR ROAD SAFETY

Road safety target can be defined as quantified road safety goal with an explicit quantitative limit and time frame. Road safety targets first appeared in the early 1970s in countries like Japan, Finland, and France.

There are two different approaches to setting targets. The top-down or idealistic approach is based on aspirational standards as Canada's vision of having the safest roads in the world or Sweden's ‘Vision Zero,” which is about placing priority on preventing death and serious injury, with little prior consideration being given to how it could be achieved. Conversely, the target can be based on a more realistic objective, through a bottom-up process, where the basis for the target is the estimated effect of the available road safety measures. The latter approach, based on research and analysis, has been adopted by countries like Australia, New Zealand, the United Kingdom, and a few other countries. In fact, many countries use a combination of the two approaches, combining idealism and realism. On the one hand, the targets must have a certain degree of public support while, on the other hand, they must have a certain degree of ambition to initiate efficient actions.

Road safety targets should be realistic and achievable, understandable, owned, based on facts, cost-effective and monitored. The existence of targets and targeted road safety programs increases the likelihood that safety policies will be implemented. Institutions in those countries with targeted road safety programs change their behavior once such a program is introduced. Targeted road safety programs can result in better integration of existing institutional efforts, generally require greater co-ordination and often produce a more focused allocation of resources.

## TARGETS FOR DEVELOPING COUNTRIES

It is important to note that developing countries should not blindly set casualty reduction targets of the type typically being set in industrialized countries. These tend to be statements such as, “Reduction in 1993 number of deaths by 30% by the year 2000.”[8] Most industrialized countries are now at the mature stage of motorization and, in many cases, approaching or even exceeding theoretical saturation levels. Consequently, their vehicle fleets are now stabilized, or at worst, are growing slowly and accident levels too have stabilized. Therefore, they can achieve reduction, with reasonable confidence set targets for casualty reductions target of 30% or more within 6-15 years and many have done so (e.g., Australia, Denmark, Netherlands, New Zealand, and UK).

Many developing countries are still approaching or just entering the explosive growth phase of motorization. Thus, private vehicle fleets will increase substantially in future years. During early stages of motorization, rapid increases in vehicle fleets inevitably result in accompanying increases in number of road accident deaths or injuries due to the populations' greater exposure to risk. Adopting a target such as ‘30% reduction on 1995 level deaths by 2000’ may in reality require reduction of 80% or more in the likely numbers of deaths that will occur by the year 2000, since actual deaths may rise by an additional 50% between 1995 and 2000 as a result of the previously mentioned increasing risk exposure.

It is also important to establish behavior-related and other targets such as “percentage of drivers wearing seat belts to increase by xx percent,” “kilometers of pedestrian footpaths increase by yy percent,” or “number of pedestrian crossing facilities increase by zz percent,” all by a set date.

## PROBLEM ANALYSIS

Road traffic accident data analysis is an important tool for determining the main safety problems toward which measures should be directed. It is important to analyze trends in fatality crashes over a longer-time span, as well as trends for less serious crashes. Also a separate review of urban and rural areas and of problem areas in the road network is useful. It is also important to analyze exposure data and risk data. Several countries gather information on traffic behavior, road user knowledge, skills, and opinions. However, data on these variables are lacking in India.

When planning traffic safety measures, an analysis of the present situation is not always sufficient; safety experts should attempt to prepare for the future. For example, in several countries the aging of the population, the growing economy, and the rising volume of traffic pose new challenges for traffic safety analysis. On the other hand, new technology offers opportunities for speed restriction, traffic management, and mitigation of collision consequences.

## ROAD SAFETY POLICIES AND STRATEGIES

Traffic safety measures are aimed at people, vehicles, the road, and its environment. In the planning process, all three elements should be considered. The areas where measures are planned and implemented are: land-use planning, transport systems and environment, traffic education, information, traffic control, telematics, and vehicle technology. Problem-solving calls for co-operation among different sectors.

The planning of safety measures is based on decreasing exposure and risks. This requires information on the influence of the measures. At the international level, a great deal of research exists on the efficiency of traffic safety measures.

The proposals for measures may involve research data collection, information on collisions, and organizing traffic safety work. These are so-called indirect measures that can be categorized separately in the program. The plan can be based on knowledge of organizational effectiveness (resulting from process evaluations, etc.). During the planning process, the options to implement, organize and finance the program at the regional and local levels must be considered. To commit various organizations to the work, officials from different sectors should be involved in the work from the outset.

## ROAD SAFETY POLICIES IN INDIA

India, up to date, does not have any proper and formal national road safety policy despite the fact that it almost tops the world as far as the total number of road fatalities is concerned. The National Road Safety Council (NRSC) ever since its inception as a consequence of MVA 1988, has tried to formulate and finalize a road safety policy for India, but without much fruitful results.

A fresh attempt to develop road safety policy was initiated by Ministry of Highways and Transport, G.O.I. at the instance of World Bank in 2003. Consultants were appointed to assist the Ministry to finalize the road safety policies and strategies. In the workshops held in connection with the project and in the Presentation made at Annual Session of Indian Roads Congress in Ahmedabad in 2004, it was learnt that the Ministry was deliberating on 11-elements for road safety policies under the vision ‘Safer Roads For Everyone.’ The first three policies deal with overall framework for road safety at the National level and include ‘Raising awareness about road safety issues among decision makers, citizens, and road users,’ ‘Providing enabling legislature, institutional, and financial base for road safety,’ and ‘Development of comprehensive road safety information database.’ The second set of policies, dealing with components of the road traffic system, include four elements and relate to safety policies for ‘Road infrastructure,’ ‘Vehicles,’ ‘Drivers,’ and ‘VRUs.’ The final group of safety policies, connected with important support systems for road safety, include four elements. viz., ‘Road safety education and training for special groups such as school children and various road safety professional groups,' ‘Enforcement,’ ‘Emergency medical services,’ and ‘HRD and research.’ It is hoped these policy statements will be adopted as National Road Safety Policy so as to serve as guidelines for road safety planning and programming at National, state, and local levels.

Planning and implementing road safety programs on scientific basis and monitoring and evaluating those requires proper institutional, legal, and funding mechanisms. India's case is more unique as the existing NRSC under the chairmanship of the Minister in-charge of Road Transport and Highways at the Center is an overall high-level advisory body. It is neither a professional nor executive body dealing with road safety issues on a regular and continuing basis. So is the case with Transport Development Council (TDC). Although TDC meets generally once in a year, the NRSC has met only seven times during last 15 years. This is the reason, perhaps, the Working Group on Road Accident, Injury Prevention, and Control (RAIPC) appointed by the Planning Commission, Government of India, 2001,[6] in its report recommended for a high-level professional body like a National Road Safety Board (NRSB), to be headed by a part-time Chairman of the rank of Cabinet or Minister of State level may also be set-up within the administrative jurisdiction of MoRTH to advice and guide road safety-related activities, including research on a regular and continuing basis, and also ‘have advantage of easier coordination in work.’ However, due to strong bureaucratic inertia the proposal did not find favor with the Road Transport Ministry officials and it was unfortunately, made sure that the same was not accepted by the Planning Commission.

The existing NRSC suffers from certain pitfalls and is not in a position to attain the stated objectives in coordinating the road safety activities. The institution can be refashioned, responding to the needs and objectives, in desirable direction, but the human beings constituting NRSC cannot always be engineered. The focus must remain on keeping NRSC proactive for the cause of road safety development. Such a body has been responsible and effective for road safety activities elsewhere in France, Japan, Republic of Korea, and some other countries, where the Prime Minister chaired such high-level council meetings, and other empowered committees constituted at lower operational level to implement the decisions of high powered apex body like NRSC.

## CONCLUSIONS

Using roads is necessary for participation in the society. However, risk of death or injury while using the roads is disproportionate, as is evident from various analyses on the basis of global road accident data. Road accident statistics also reveal that poor and developing countries are bearing the brunt of the road safety problem, despite low levels of motorization. Still more unfortunate part is that India stands out well above other developing and developed nations with regard to road safety situation, rather in an uncomplimentary way. Things are getting bad to worse each year and yet nothing significant seems to be happening to improve road safety situation in the country. India seems to be almost stuck at the threshold of the third and the final phase of the road safety development process, which other countries have gone through and benefited. Road safety is being treated in a laissez faire way in India. Among the important reasons for such a situation are the lack of national commitment to the road safety, bureaucratic inertia, and public apathy to human suffering and economic losses due to road accidents.

The industrialized countries in many parts of the world-treated accidents as a man-made and public health problem and implemented major comprehensive and interdisciplinary programs over the last quarter century. However, the accident problem in India have not yet been taken up in that perspective. The very limited actions to tackle the problem by various public agencies have been independent of any well-thought and well-researched road safety policy and associated action plans, and therefore had, as expected, poor outcomes.

Fortunately, many of these deaths and injuries on roads are preventable and affordable as the experience of many countries show. The countries that have been most successful in reducing the number of crashes have many commonalities; the main factors include a political commitment, a broad strategy, a clear vision, a concrete plan, institutional development and co-ordination, and a careful and critical evaluation of measures and their effectiveness. Adoption of an appropriate Road Safety Policy and Road Safety Action Plans was the main driving force as it stimulated effective and efficient countermeasures to the identified problem areas and enabled all relevant parties to deliver their contributions in a timely and co-operative manner. Despite the fact that India tops the world as for as the total number of road fatalities is concerned, it does not have any proper and formal national road safety policy as yet.

We know deaths due to road accidents can be drastically reduced, but we will have to convince decision-makers, stakeholders, and the public at large that the present framework of road safety in the country needs drastic improvement and up-gradation. It is only with suitable institutional, financial, and legal framework that we may be able to see proper road safety policies and action plans on the ground for increased road safety. Road deaths can be drastically reduced if enough people and interests are convinced that they want it to happen and are willing to work together to make it happen without any prejudices and vested interests.
